# *Streptococcus iniae* M-Like Protein Contributes to Virulence in Fish and Is a Target for Live Attenuated Vaccine Development

**DOI:** 10.1371/journal.pone.0002824

**Published:** 2008-07-30

**Authors:** Jeffrey B. Locke, Ramy K. Aziz, Mike R. Vicknair, Victor Nizet, John T. Buchanan

**Affiliations:** 1 Department of Pediatrics, University of California San Diego, La Jolla, California, United States of America; 2 Center for Marine Biotechnology & Biomedicine, Scripps Institution of Oceanography, University of California San Diego, La Jolla, California, United States of America; 3 Department of Microbiology and Immunology, Faculty of Pharmacy, Cairo University, Cairo, Egypt; 4 Kent SeaTech Corporation, San Diego, California, United States of America; 5 Skaggs School of Pharmacy & Pharmaceutical Sciences, University of California San Diego, La Jolla, California, United States of America; 6 Aqua Bounty Technologies, San Diego, California, United States of America; Centre for DNA Fingerprinting and Diagnostics, India

## Abstract

**Background:**

*Streptococcus iniae* is a significant pathogen in finfish aquaculture, though knowledge of virulence determinants is lacking. Through pyrosequencing of the *S. iniae* genome we have identified two gene homologues to classical surface-anchored streptococcal virulence factors: M-like protein (*simA*) and C5a peptidase (*scpI*).

**Methodology/Principal Findings:**

*S. iniae* possesses a Mga-like locus containing *simA* and a divergently transcribed putative *mga*-like regulatory gene, *mgx*. In contrast to the Mga locus of group A *Streptococcus* (GAS, *S. pyogenes*), *scpI* is located distally in the chromosome. Comparative sequence analysis of the Mgx locus revealed only one significant variant, a strain with an insertion frameshift mutation in *simA* and a deletion mutation in a region downstream of *mgx*, generating an ORF which may encode a second putative *mga*-like gene, *mgx2*. Allelic exchange mutagenesis of *simA* and *scpI* was employed to investigate the potential role of these genes in *S. iniae* virulence. Our hybrid striped bass (HSB) and zebrafish models of infection revealed that M-like protein contributes significantly to *S. iniae* pathogenesis whereas C5a peptidase-like protein does not. Further, *in vitro* cell-based analyses indicate that SiMA, like other M family proteins, contributes to cellular adherence and invasion and provides resistance to phagocytic killing. Attenuation in our virulence models was also observed in the *S. iniae* isolate possessing a natural *simA* mutation. Vaccination of HSB with the Δ*simA* mutant provided 100% protection against subsequent challenge with a lethal dose of wild-type (WT) *S. iniae* after 1,400 degree days, and shows promise as a target for live attenuated vaccine development.

**Conclusions/Significance:**

Analysis of M-like protein and C5a peptidase through allelic replacement revealed that M-like protein plays a significant role in *S. iniae* virulence, and the Mga-like locus, which may regulate expression of this gene, has an unusual arrangement. The M-like protein mutant created in this research holds promise as live-attenuated vaccine.

## Introduction

*Streptococcus iniae* is a significant finfish pathogen responsible for annual losses in aquaculture exceeding $100 million [1]. Though originally isolated from a freshwater Amazon dolphin (*Inia geoffrensis*) [2], and capable of causing infection in elderly or otherwise immunocompromised humans [3], *S. iniae* is predominantly a fish pathogen with a broad host range of fresh and saltwater species such as trout, tilapia, salmon, barramundi, yellowtail, flounder, and hybrid striped bass (HSB) [4]. Mortality resulting from *S. iniae* is often attributed to meningoencephalitis which manifests following systemic dissemination of bacteria through the bloodstream and major organs [4]. Currently there are no commercial vaccines approved for prevention of *S. iniae* infection in US aquaculture.

Our understanding of *S. iniae* pathogenesis is limited. To date only three *S. iniae* virulence factors have been characterized in the context of fish virulence: the capsular polysaccharide which contributes to phagocyte resistance [5], [6]; the cytolysin streptolysin S which contributes to host cell injury [7], [8]; and phosphoglucomutase, which is required for cell wall rigidity and resistance to cationic antimicrobial peptides [9]. In each case, the identified *S. iniae* virulence determinant shared homology with counterparts expressed by other major streptococcal pathogens of humans and/or animals. In an effort to identify additional genes involved in *S. iniae* pathogenesis, we have used pyrosequencing [10] (454 Life Sciences) of a virulent isolate to identify candidate genes sharing homology with proven virulence factors of the leading human pathogen, *Streptococcus pyogenes* (group A *Streptococcus*, GAS), a well characterized close genetic relative of *S. iniae* [11].

In GAS, many virulence genes are part of a pathogenicity regulon known as Mga (multiple gene regulator of group A *Streptococcus*) [12], [13]. Mga is a “stand-alone” global gene regulator that exerts positive transcriptional regulation on downstream genes in the proximal Mga locus, and distally in the genome through binding of the Mga protein to consensus upstream promoter regions [14], [15]. The most extensively studied component of the Mga regulon is M protein, a surface-anchored virulence factor [16], [17] that contributes to GAS cellular adherence and invasion [18], [19], resistance to phagocytic clearance [20], [21], host inflammatory activation [22], [23], and serotypic diversity [24], [25]. Other members of the GAS Mga regulon include genes for additional M-like surface proteins and the gene encoding the C5a peptidase ScpA, a bifunctional virulence factor capable of inactivating the complement derived neutrophil chemoattractant C5a [26], [27], while also contributing to GAS epithelial cell adhesion [28].

Here we identify genes *simA* and *scpI* in a virulent *S. iniae* isolate which share homology with genes encoding the GAS Mga-associated virulence factors M-like protein and C5a peptidase, respectively. We provide bioinformatic analyses of these two genes and the *S. iniae* Mga-like Mgx locus, comparing different *S. iniae* isolates and other streptococcal pathogens. Through targeted allelic replacement mutagenesis coupled with *in vitro* and *in vivo* models of *S. iniae* pathogenesis, we assess the roles of these genes as virulence determinants of this leading aquaculture pathogen, and demonstrate a key role for *simA*. Finally, we examine the utility of the Δ*simA* mutant as a live attenuated vaccine.

## Results

### SiMA and its relationship to other streptococcal M family proteins

**Figure 1:**
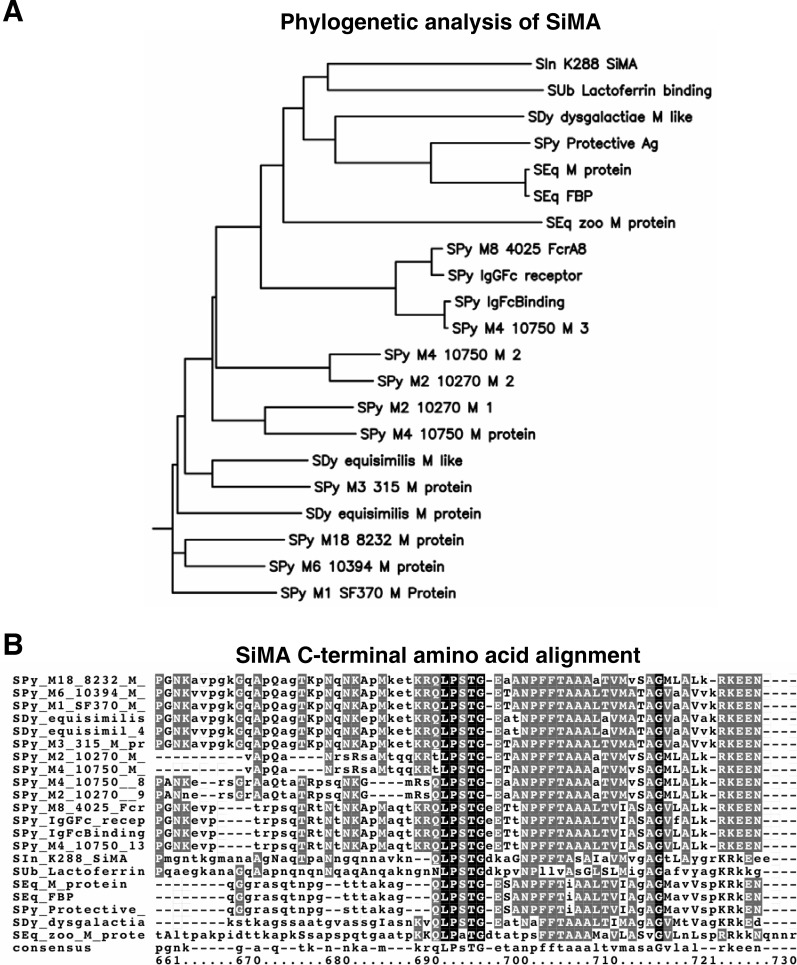
Bioinformatic analysis of SiMA. (A) Phylogenetic clustering of SiMA shows greatest similarity to other streptococcal M family proteins, most closely the *S. uberis* lactoferrin binding protein. (B) Amino acid sequence alignments of SiMA with other streptococcal M family proteins shows highest conservation in the C-terminal region which includes the LPXTG surface anchor motif. Strain abbreviations: SIn–*S. iniae*, SPy–*S. pyogenes*, SUb–*S. uberis*, SEq–*S. equi*, and SDy–*S. dysgalactiae*.

The 1,566 bp M-like protein gene *simA*, from *S. iniae* strain K288, encodes a 521 amino acid gene product, SiM (*S. iniae* M-like protein), with a predicted precursor protein mass of 57.5 kDa. This M-like protein gene is identical to the recently published *simA* gene sequences from *S. iniae* strains QMA0076 and QMA0131 [29]. BLAST (tblastn) analysis groups SiMA closest to the *S. uberis* lactoferrin binding protein, Lbp (32% identity, 49% positive) [30] and the *S. dysgalactiae* subsp. *dysgalactiae* (GCS) M-like protein, DemA (31% identity, 51% positive) [31], though SiMA has near comparable similarity to a number of other streptococcal M family proteins (Fig. 1A). Amino acid sequence alignments between SiMA and related M family proteins, as expected, showed the highest degree of similarity in the C-terminus which includes the LPXTG Gram-positive surface anchor motif (Fig. 1B, S1) [32].

### *sim* sequences are highly conserved across a diverse panel of *S. iniae* isolates

The *sim* genes from a panel of 11 *S. iniae* isolates from various hosts and geographical regions in North America were analyzed for DNA sequence similarity (Table 1). Only three of these strains (29178, 95006, and 02161A) varied from the *simA* consensus sequence defined in the wild-type (WT) K288 strain, a finding consistent with previous observations [29]. ATCC strain 29178 (freshwater dolphin abscess isolate) possesses a silent A→G single nucleotide polymorphism (SNP) in nucleotide 741, maintaining the Gln-247 residue, and is identical to the *simA* allele sequence for the QMA0140 dolphin isolate [29]. Another A→G SNP was found in strain 95006 (tilapia abscess isolate) at nucleotide 1,430, changing Gln-477 to Arg-477. The most significant *sim* sequence variation was found in a tilapia brain isolate (02161A), which possess a 40 bp insertion duplication starting at bp 595. This insertion generates a frameshift mutation splitting the gene into two potential ORFs, likely leading to severely altered or absent function. The first ORF is predicted to encode a truncated N-terminal SiM fragment of predicted 22.7 kDa mass, but would lack the LPXTG consensus motif for sortase-mediated cell wall anchoring of Gram-positive surface proteins. The second ORF would encode a C-terminal SiM fragment of 33.7 kDa containing the LPXTG motif, but would lack the hydrophobic N-terminal leader sequence involved in protein secretion.

**Table 1:** Information on *S. iniae* strains used in *sim* gene sequencing.

Strain	Source	Location	Host	Tissue origin	Reference
K288	KST	California	HSB	brain	[9]
K139	KST	California	HSB	brain	
K436	KST	California	HSB	brain	
94290	KST	California	HSB	internal organs	
94426	LSU	Louisiana	tilapia	brain	[5]
95006	LSU	Louisiana	tilapia	abscess	
94449	LSU	Louisiana	tilapia	abscess	
9117	UT	Ontario	human	blood	[3]
9066	UT	Ontario	fish (sp. unknown)	surface of skin	[100]
F1	UF	Florida	rainbow shark	systemic	[101]
29178	ATCC	San Francisco	freshwater dolphin	abscess	[2]
02161A	LSU	Minnesota	tilapia	brain	

Abbreviations: KST–Kent SeaTech Corporation, HSB–hybrid striped bass, LSU–Louisiana State University, UT–University of Toronto, UF–University of Florida, ATCC–American Type Culture Collection.

### *ScpI* and its relationship to other streptococcal C5a peptidase-family genes

The *S. iniae scpI* (Streptococcal C5a peptidase-like gene of *S. iniae*) gene is 3,369 bp in length and encodes a predicted 1,122 amino acid gene product with a mass of 123.3 kDa. BLAST (tblastn) analysis indicates ScpI has equal degrees of similarity (37% identity, 55% positive) to the C5a peptidases of GAS (ScpA of the Manfredo M5 strain) [33] and group B *Streptococcus* (*S. agalactiae*, GBS) (ScpB of the A909 strain) [34]. Though the proteolytic functionality of ScpI is unknown, it does contain the conserved serine protease catalytic triad of Asp-130, His-193, and Ser-512 [35]; however due to differences in overall protein size these conserved residues fall at slightly different locations in ScpI (Asp-114, His-181, Ser-501) (Fig. S2). Analysis of ScpI also indicates conservation of the C-terminal LPXTN cell surface anchor motif (Fig. S2).

### *S. iniae* does not possess a GAS-like Mga locus

**Figure 2:**
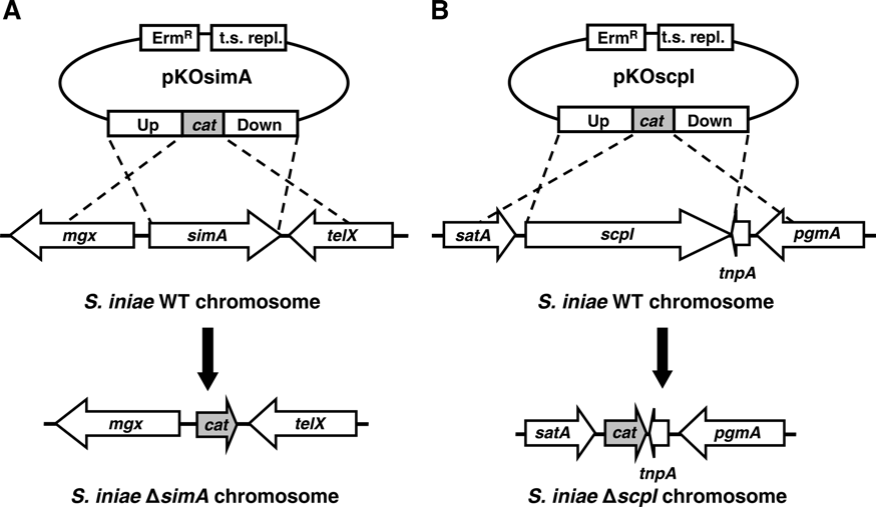
Allelic exchange mutagenesis of *simA* and *scpI*. Allelic exchange mutagenesis of *simA* (A) and *scpI* (B) was carried out by using knockout plasmids (pKOsimA and pKOscpI) containing ∼1,000 bp flanking regions upstream (Up) and downstream (Down) nesting the *cat* gene in between. The plasmid also contains Erm resistance (Erm^R^) and a temperature-sensitive origin of replication (t.s. repl.). Through two independent single crossover events, the *S. iniae simA* and *scpI* genes were precisely replaced in-frame by the *cat* gene. (A) The *simA* gene is located adjacent to a putative *mga*-like regulatory gene, *mgx*. Downstream is a divergently transcribed, putative tellurite resistance protein (*telX*). (B) The *scpI* gene lies upstream from a putative sugar ABC transporter gene (*satA*). A putative transposase (*tnpA*) flanks the downstream end of *scpI* followed by the phosphoglucomutase gene (*pgmA*).

*S. iniae* does not possess a typical GAS-like Mga locus arrangement containing M family protein and C5a peptidase genes, where these genes in GAS are located adjacently and downstream of the *mga* gene transcribed in the same direction [15]. Unlike GAS, in *S. iniae* strain K288, the M-like protein gene (*simA*) is located adjacent to a divergently transcribed *mga*-like gene, *mgx* (Fig. 2A) and the C5a peptidase gene (*scpI*) is located elsewhere on the chromosome. The Mga-like Mgx shares almost complete amino acid similarity (98.2% identity, 98.4% positive) with the Mgx sequence reported for *S. iniae* strain QMA0076 [29]. The limited sequence variation is isolated to the C-terminal amino acids leading up to and including 7 additional amino acids found in the Mgx proteins of strains K288 and 9117 (a human isolate currently being sequenced by Baylor College of Medicine Human Genome Sequencing Center, BCM-HGSC) which extend beyond the 495 amino acid Mgx protein found in strains 02161A and QMA0076. *S. iniae* Mgx is most similar (tblastn, 39% identity, 58% positive) to the Mga-like Mgc putative regulatory protein of *S. dysgalactiae* subsp. *equisimilis* (GCS/GGS) [36].

**Figure 3:**
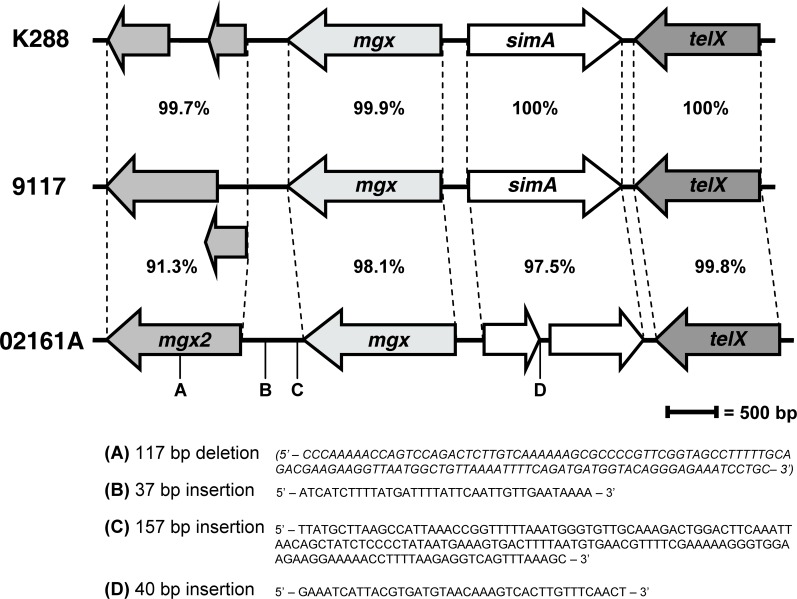
Nucleotide and ORF variability in the *S. iniae* Mga-like Mgx region. The *S. iniae* putative *mga*-like gene *mgx* and the putative tellurite resistance gene *telX* are highly conserved in strains K288, 9117, and 02161A. 02161A, however, has significant variation in the Mgx chromosomal region primarily due to four deletion or insertion sequences (A–D), two of which affect coding sequences. A 40 bp insertion/duplication (D) in the *simA* M-like protein gene splits it into two ORFs whose transcription and function is unknown. A 117 bp deletion (A) in the upstream *mgx* region generates a second putative *mga*-like gene, *mgx2*. In K288 and 9117 the mgx2 region is broken into two smaller ORFs. Similarity between adjacent strains is indicated as % nucleotide identity.

Our sequencing efforts, as well as the BCM-HGSC 9117 genome project, indicate the presence of a chromosomal region downstream from *mgx* which encodes two ORFs with high BLAST similarity to regions of Mgx and other Mga-like regulatory proteins, potentially representing an evolutionary distant duplication of a *mga*-like gene, whose function was lost through mutations over time (Fig. 3). Strain 02161A, however, through sequence variation in this region, including a 117 bp deletion, possesses a 1,326 bp ORF which may encode a second putative *mga*-like regulatory gene, *mgx2* (Fig. 3). The 441 amino acid *mgx2* gene product, Mgx2, has a predicted mass of 51.7 kDa and is most similar (tblastn) to the Mgx protein of *S. iniae* QMA0076 (42% identity, 58% positive) [29], the DmgB Mga-like protein of GCS strain Epi9 (32% identity, 52% positive) [31], and the Mga protein of GAS strain MGAS8232 (32% identity, 53% positive) [37].

**Figure 4:**
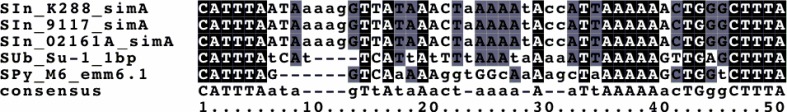
Comparison of putative Mga-like binding motifs upstream of *sim* genes. The 51 bp upstream regions of *S. iniae sim* genes with high similarity to GAS *emm* gene Mga binding sites are identical in strains K288, 9117, and 02161A. A 47 bp sequence sharing similarity to Mga-like binding sites located upstream of the gene encoding the *S. uberis* lactoferrin binding protein (Lbp, a close phylogenetic relative of SiMA) is also included for comparison. *S. iniae* and *S. uberis* putative binding motifs are aligned with the established 45 bp Mga binding site found in M6 GAS upstream of the *emm6.1* M protein gene. Abbreviations: SIn–*S. iniae*, SUb–*S. uberis*, SPy–*S. pyogenes*.

Almost exactly halfway between the divergently transcribed *mgx* and *simA* genes (162 bp upstream from the *simA* start codon ) lies a highly conserved 51 bp region, identical in isolates K288, 9117, 02161A and QMA00131 [29] (Fig. 4). This region has similarity to the established 45 bp Mga binding site for the *emm6.1* gene of M6 GAS [14] and a 47 bp region upstream of the *S. uberis* lactoferrin binding protein gene [30] (Fig. 4). Downstream from *simA* is a putative tellurite resistance protein gene, *telX*, encoding a gene product with 99% identical amino acid composition to the TelX protein of *S. iniae* strain QMA0076 [29]. The chromosomal arrangement of *mgx*, *simA*, and *telX* was identical in *S. iniae* strains K288, 9117, 02161A, and QMA0076 [29]. Aside from insertion and deletion mutations in 02161A, nucleotide level analysis of the remainder of the Mgx locus reveals high conservation between strains (Fig. 3).

Unlike GAS, but similar to the chromosomal positioning in GCS and GGS [36], the *S. iniae scpI* gene is located outside of the Mga-like locus of the chromosome (Fig. 2B) and does not possess an upstream promoter region similar to binding motifs present in Mga-regulated GAS *scpA* genes [14]. *ScpI* is bordered downstream by a divergently transcribed putative transposase (*tnpA*), a 237 bp ORF encoding a 78 amino acid gene product. TnpA has highest similarity (tblastn, 66% identity, 78% positive) within an overlapping 50 amino acid region of “IS861, transposase orfB” in the GBS A909 genome [34]. A transposase is one of the insertional elements flanking the GBS *scpB* chromosomal region and is thought to be involved in horizontal gene transfer [38]. Immediately upstream of the transposase is the phosphoglucomutase gene (*pgmA*) which has been implicated in *S. iniae* fish virulence [9]. Upstream of *scpI* lies a 957 bp putative sugar ABC transporter gene (*satA*) with high similarity (tblastn, 90% identity, 96% positive) to the putative ABC sugar transporter SPy_1225 of GAS M1 strain SF370 [39]. The presence of *scpI* and *tnpA* in between the *satA* and *pgmA* genes in the *S. iniae* chromosome also supports horizontal transfer theories since the homologues of *satA* and *pgmA* in GAS are located adjacently in the genome [39].

### Allelic replacement of *simA* and *scpI* conserves key *S. iniae* phenotypic properties

**Figure 5:**
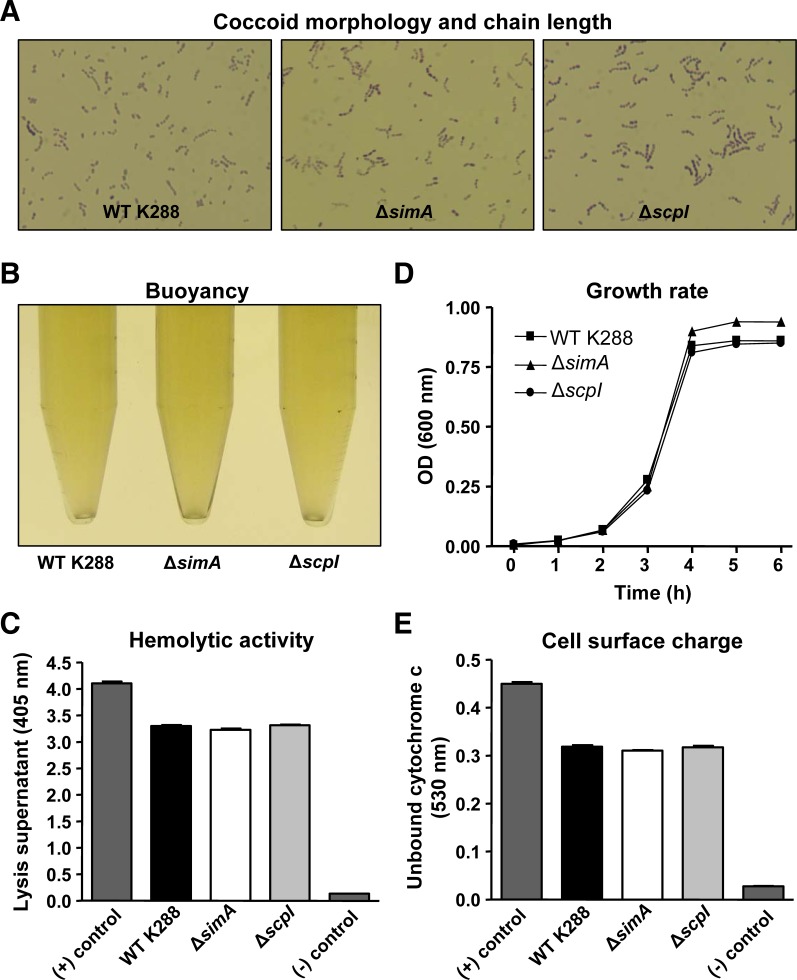
Basic phenotypic properties of *S. iniae* are highly conserved following allelic replacement of *simA* and *scpI*. (A) Cocci chain morphology was observed under light microscopy (Crystal Violet staining viewed under an oil immersion 100× objective). (B) General buoyancy characteristics of the strains were observed in overnight cultures grown in 15 ml conical tubes. (C) Hemolytic activity was measured through the optical density of the supernatant following incubation of HSB red blood cells with bacteria. (D) Growth rate was measured optically every 45 min in 5 ml tube cultures. (E) Bacterial cell surface charge was indirectly measured through the absorbance of unbound, positively charged cytochrome *c*, following incubation with bacteria.

Precise in-frame allelic replacement of *simA* and *scpI* (Fig. 2A, B) generated viable mutants which retain most WT phenotypic characteristics. In particular, no differences between the Δ*simA* or Δ*scpI* mutant and the WT K288 parent strain were observed in coccoid morphology (Fig. 5A), cell buoyancy which is correlated to encapsulation (Fig. 5B), hemolytic activity against fish red blood cells (Fig. 5C), or cell surface charge (Fig. 5E). The Δ*simA* mutant did enter stationary phase at a slightly higher optical density than either the WT K288 or the Δ*scpI* mutant (Fig. 5D) and the Δ*scpI* mutant had a slightly increased frequency of multimeric cocci chains than the other two strains (Fig. 5A).

### *S. iniae* M-like protein contributes to virulence in HSB and zebrafish infection models

**Figure 6:**
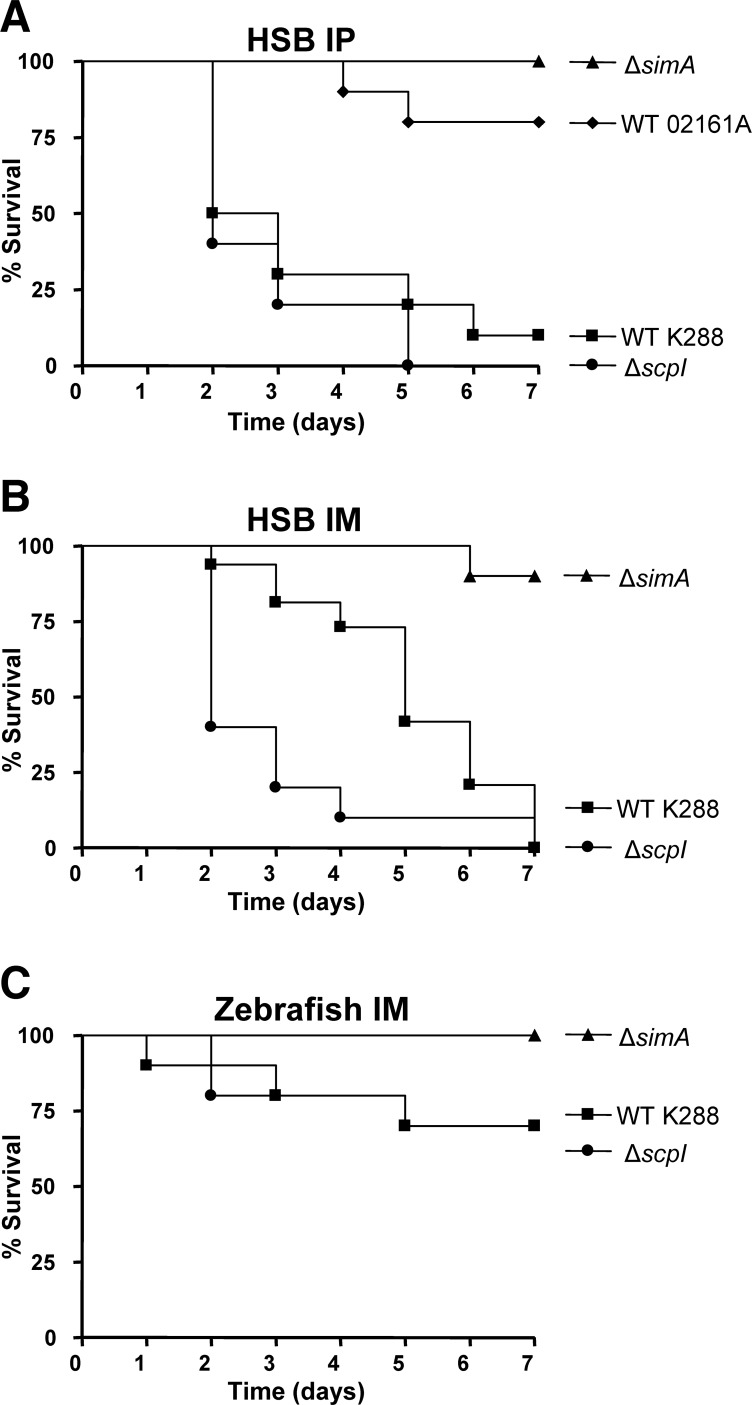
M-like protein contributes to *S. iniae* virulence in HSB and zebrafish infection models. (A) Juvenile hybrid striped bass (HSB) (n = 10) were injected IP with 3×10^5^ CFU of WT K288 *S. iniae*, the Δ*simA* and Δ*scpI* isogenic mutants, or WT 02161A (possesses a natural frameshift mutation in *simA*). (B) Juvenile HSB (n = 10) were injected IM with 3×10^5^ CFU of WT K288 *S. iniae*, or the Δ*simA* and the Δ*scpI* mutants. (C) Adult zebrafish (n = 10) were injected IM with 4×10^5^ CFU of WT K288 *S. iniae* or the Δ*simA* and the Δ*scpI* mutants.

Using our established *S. iniae* HSB infection model system [9] we analyzed the overall requirement of *simA* and *scpI* for fish virulence following intraperitoneal (IP) or intramuscular (IM) challenge. Compared to the WT K288 strain, the isogenic Δ*simA* mutant was completely attenuated in the HSB IP challenge (*P*<0.0001) (Fig. 6A) and caused only 10% mortality in the IM challenge group (*P*<0.001) (Fig. 6B). An IP challenge in HSB with 1,000 times the lethal WT K288 dose (3×10^8^ CFU) of the Δ*simA* mutant was required to generate comparable mortality to WT K288 (data not shown). Similar to the K288 Δ*simA* mutant, *S. iniae* WT 02161A strain (with a frameshift mutation truncating the *simA* ORF) was attenuated compared to strain K288 in the HSB IP challenge model (*P*<0.005) (Fig. 6A). In contrast, allelic replacement of the *scpI* gene encoding a C5a peptidase-like protein did not significantly reduce *S. iniae* virulence in the IP model (*P* = 0.31) (Fig. 6A) and was actually associated with an increase in the kinetics of killing compared to WT K288 in the IM challenge model (*P*<0.01) (Fig. 6B). A zebrafish IM challenge model has also been developed for analysis of virulence factors of streptococcal pathogens [40], including the observed attenuation of a GAS C5a peptidase (ScpA) mutant compared to its parent strain [41]. We found that the *S. iniae* Δ*simA* mutant showed evidence of attenuation in this zebrafish model, producing no mortalities, though this trend did not achieve statistical significance due to low WT mortalities (*P* = 0.067). Challenge with the isogenic Δ*scpI* mutant generated no evidence of attenuation and a similar mortality curve to the WT K288 *S. iniae* parent strain in the zebrafish model (*P* = 0.985) (Fig. 6C). Based on the composite *in vivo* fish challenge experiments, we conclude *S. iniae* M-like protein SiMA plays a significant role in *S. iniae* invasive disease pathogenesis, while the C5a peptidase-like protein ScpI alone is not required for fish virulence upon systemic challenge by injection.

### SiMA does not protect *S. iniae* against cationic AMPs

AMPs are an evolutionarily conserved innate defense mechanism [42], and likely play a role in fish resistance to bacterial infection [43]. The increased sensitivity of an *S. iniae* phosphoglucomutase mutant to cationic AMPs demonstrates the importance of *S. iniae* to protect against antimicrobial defenses [9]. To determine if enhanced AMP resistance represent a contribution of SiMA to *S. iniae* virulence, we tested the susceptibility of the Δ*simA* mutant to three AMPs: *Bacillus*-derived polymyxin B, HSB derived-moronecidin, and murine-derived CRAMP. Both WT and Δ*simA* mutant *S. iniae* strains were sensitive to all three AMPs and killed with similar efficiency: 99.10±0.03% WT vs. 99.38±0.03% Δ*simA* killing by 60 µM polymyxin B in 120 min; 99.21±0.06% WT vs. 98.74±0.14% Δ*simA* killing by 1.5 µM moronecidin in 15 min; 99.98±0.05% WT vs. 99.92±0.47% Δ*simA* killing by 16 µM CRAMP in 30 min. We conclude that SiMA does not likely contribute to relative resistance of *S. iniae* to cationic AMPs.

### SiMA contributes to *S. iniae* adherence to and invasion of fish epithelial cells

**Figure 7:**
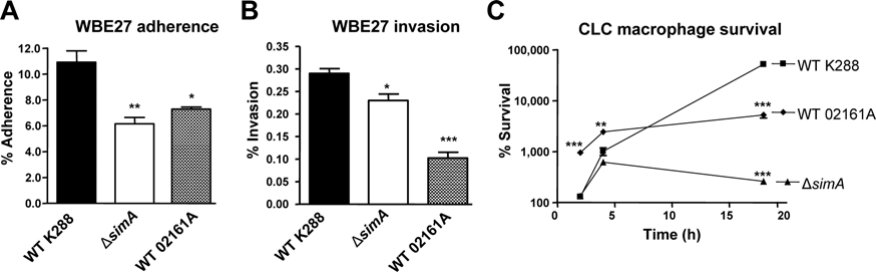
M-like protein contributes to *S. iniae* adherence to and invasion of cultured fish epithelial cells and resistance to killing by fish macrophages. (A) Adherence and (B) invasion characteristics of WT K288, the isogenic Δ*simA* allelic mutant, and the naturally M-deficient WT 02161A *S. iniae* strain for the fish epithelial cell line WBE27. (C) Survival of WT K288, WT 02161A, and the Δ*simA* mutant upon co-incubation with CLC fish macrophage/monocytes for 2, 4, or 18 h. Significance indicated as: * *P*<0.05, ** *P*<0.005, *** *P*<0.0005. Data are presented as mean±SEM from two-tailed t-tests.

The ability to adhere to and invade epithelial layers is proposed to play a role in *S. iniae* virulence [44]. We used cultured monolayers of the white bass epithelial cell line WBE27 to assess the adherence and intracellular invasive properties of *S. iniae* strains *in vitro* [45]. Compared to the WT parent strain K288, the *S. iniae* Δ*simA* mutant demonstrated significantly less adherence to (∼40% reduction, *P*<0.005) and invasion (∼20% reduction, *P*<0.02) of WBE27 cells (Fig. 7A, B). The levels of adherence and invasion associated with the *S. iniae* WT 02161A strain (harboring a frameshift/truncation mutation in the *simA* gene) had a similar trend (*P* = 0.0067, *P*<0.0001, respectively) to those of the *S. iniae* K288 Δ*simA* mutant (Fig. 7A, B).

### SiMA contributes to *S. iniae* macrophage resistance

Another described virulence property of *S. iniae* is its ability to resist phagocytosis and survive within fish leukocytes [46]. To determine if M-like protein SiMA promotes bacterial survival when exposed to phagocytic cells, a killing assay with the carp macrophage cell line CLC was performed. The survival of the Δ*simA* mutant in the presence of macrophages was similar to that of the parent strain at early time points (2 h and 4 h), however by 18 h survival of the Δ*simA* mutant was reduced over 2 logs compared to WT K288 (*P*<0.0001) (Fig. 7C). The WT 02161A strain possessing the frameshift/truncation mutation in the *simA* gene also showed significantly diminished survival compared to WT strain K288 by the 18 h time point (*P*<0.0001) (Fig. 7C).

### The attenuated Δ*simA* mutant confers adaptive immune protection against *S. iniae* infection

Previous work demonstrated the effectiveness of an attenuated *S. iniae* phosphoglucomutase mutant to protect HSB against subsequent challenge with WT *S. iniae* [9]. Because the Δ*simA* mutant shows significant attenuation in our HSB infection challenges, we investigated its potential to serve as a live attenuated vaccine. IP vaccination of HSB with two different doses of the Δ*simA* mutant (3×10^4^ and 3×10^6^ CFU) resulted in 8% mortality in each group. No mortality was observed in the PBS mock vaccination group. Following a holding period of 90 days (∼1,400 degree days), both vaccinate groups were completely protected from a lethal dose (LD_96_, 5×10^5^ CFU) of WT K288 *S. iniae* (Table 2), demonstrating the high protective capacity of this mutant as a live vaccine candidate.

**Table 2:** Immune protection conferred by the Δ*simA* mutant in HSB.

Vaccination group	% Survival: Ä*simA* vaccination	% Survival: WT K288 challenge
3×10^4^ CFU	92	100
3×10^6^ CFU	92	100
PBS control	100	4

## Discussion

To further understanding of *S. iniae* virulence, we used whole genome pyrosequencing to identify and characterize the *S. iniae* homologues of two well-established, Mga-regulated GAS virulence factors, M-like protein (*simA*) and C5a peptidase (*scpI*). We identified in *S. iniae* strain K288 a *mga*-like locus containing the M-like protein gene *simA* and a putative *mga*-like regulatory gene *mgx*, identical in arrangement to a locus recently described in *S. iniae* strain QMA0076 [29]. The GAS Mga locus contains several downstream virulence genes regulated by Mga, including genes encoding M-proteins and C5a peptidase. Though *S. iniae* does possess a putative tellurite resistance protein gene (*telX*) downstream of *sim* (which may potentially have a role in virulence) there are no typical GAS Mga locus-like candidate virulence genes in the Mgx locus aside from *sim*. Also of note is that unlike the GAS Mga locus (and Mga-like loci in GCS/GGS), *mgx* is transcribed divergently from M-protein homologue *sim*, similar to the chromosomal juxtaposition of the closely related *S. uberis* lactoferrin binding protein gene and its putative *mga*-like regulator [30]. Additionally, the C5a peptidase-like gene (*scpI*) is positioned distally on the chromosome from *simA* and *mgx*, a chromosomal arrangement more similar to that of GCS and GGS than GAS [36]. The presence of two adjacent Mga-like genes (one of which has been disrupted with mutations in some strains) is a unique property of *S. iniae* and may hold clues to the evolution of Mga-family genes in this species. Gene duplications in the Mga locus are thought to account for the diversity of GAS M family genes [47], though we have not found any reports of duplications in *mga* or *mga*-like genes. The functionality of *mgx2* in strain 02161A, and whether the mutations leading to a disrupted *simA* gene and the creation of a second putative *mgx* gene represent an alternative virulence strategy in this strain, are interesting areas for investigation. Sequence upstream of *simA* with strong similarity to the Mga transcriptional regulatory binding domains of GAS M proteins suggests that Mgx regulation of *simA* expression is likely to occur in *S. iniae*. This hypothesis is strengthened by predicted structural analysis of Mgx that indicates helix-turn-helix domains [29], a feature present in DNA-binding Mga and Mga-like regulatory proteins [48].

C5a peptidase has highly specific endoproteolytic activity against the complement system polymorphonuclear leukocyte chemotaxin C5a [26], [27], thereby altering neutrophil trafficking to the site of infection [49], [50]. C5a peptidase also acts as an adhesin in GBS through binding to host fibronectin [51], [52] and in GAS through fibronectin independent binding [28]. Allelic replacement of the *S. iniae scpI* gene encoding a predicted C5a peptidase-like surface protein did not significantly attenuate virulence in our analyses using HSB and zebrafish infection models. While Scp inhibition of leukocyte chemotaxis is well documented in other streptococci [26], [27], inactivation of this gene does not always translate into a reduction in overall *in vivo* virulence [53]–[55]. Teleosts do possess a potent complement system [56], [57], a functional C5a homologue [58], [59], and a corresponding receptor [60], [61], so it is plausible that a fish pathogen would target components of this pathway. Additionally, *S. iniae* may possess other gene encoded determinants with functional redundancy to ScpI, masking virulence effects in our challenge systems. For example, in our genomic sequence analysis we also identified a putative C3 proteinase (data not shown) which may serve to inactivate the complement system upstream of C5a peptidase.

It appears that there is not a high degree of variation among SiM proteins. Sequencing a panel of 11 diverse *S. iniae* isolates generated only one significant sequence variation. We report an insertional frameshift mutation of the *simA* gene in *S. iniae* strain 02161A that splits the coding region into two smaller ORFs of unknown function, and note that this strain is attenuated in the HSB model. In GAS, frameshift mutations followed by compensatory mutations that bring the gene back into frame are proposed to play a role in antigenic variation of M proteins [62], a scenario that may play out over time in 02161A to generate a novel *sim* allele. The only other documented *sim* allele, *simB*, was found in strain QMA0141 as part of a similar comparative analysis of *sim* sequences [29]. These findings contrast the extreme variation in allele types for M-protein in GAS [63]. The functional implications of this conservation among *sim* alleles warrant further investigation. Additional sequencing efforts are needed to gauge the degree of *S. iniae* M-like protein sequence divergence and to determine if this surface protein may contribute as a serotyping determinate.

M family proteins have been shown to play a prominent role in colonization through adherence in multiple host pathogen systems [18], [19]. In GAS, adherence mediated by M family proteins is not universal to all cell types, but has been shown to be particularly important in binding to keratinocytes [64] and Hep-2 cells [65]. The ability to invade non-immune cell types has also been linked to the GAS M protein [66]. Consistent with these roles we observed a decrease in adherence and invasion of the white bass epithelial cell line by the *S. iniae* Δ*simA* mutant.

A primary function function of M-like proteins involves resistance to phagocytic clearance mechanisms [67]–[69]. Through binding to serum proteins such as immunoglobulins, fibrinogen, and the complement regulator, factor H, M family proteins can effectively avoid phagocytosis through prevention of complement deposition. M proteins have also been shown to confer intracellular protection against phagocytic killing [21]. Similarly for SiMA, in the presence of fish macrophages, we observed over a 2 log-fold reduction in survival in the Δ*simA* mutant compared to WT K288. Our findings confirm a role for SiM in evading phagocytosis, as suggested in studies linking SiM with fibrinogen binding [29].

Our live attenuated vaccine development approach contrasts typical M protein vaccine strategies which use the protein itself or fragments thereof as the immunogen. Such vaccination strategies for the GAS M protein have required multimeric vaccines to ensure protection against a panel of relevant serotypes [70], [71]. The generation of an autoimmune response through production of cross reactive antibodies [72], [73] against M proteins that demonstrate molecular mimicry of host tissues [74], [75] has also been a significant hurdle to GAS protein based vaccine development efforts. Whether either of these is issues is a concern in for SiM is unknown, but by deleting the M-like protein from *S. iniae* and relying on other key antigenic epitopes, both of these potential issues are circumvented in our live vaccine approach. Live attenuated vaccines also offer the advantage of prolonged, unaltered antigen presentation which can stimulate a more robust humoral and cell-mediated immune response, resulting in greater adaptive immune protection compared to inactivated bacterins or subunit vaccines in fish [76]–[78]. Successful demonstrations of live vaccines have been employed for a number of bacterial finfish pathogens [79]–[81] including *S. iniae* [9]. Though limited mortality was observed in our vaccinations with the Δ*simA* mutant, further attenuation of this strain by targeted gene disruption of additional proven virulence determinants will likely be required to provide an optimal safety profile.

In sum, through sequence analysis of the *S. iniae* genome we have identified two putative homologues of classic surface-anchored streptococcal virulence determinants, M-like protein and C5a peptidase. Allelic replacement of these two genes and analyses using our models of bacterial pathogenesis revealed that M-like protein plays a significant role in *S. iniae* virulence whereas C5a peptidase-like protein does not. Future research will investigate the regulation of these genes and their specific protein-ligand interactions. The M-like protein mutant created in this research holds promise as live attenuated vaccine. Subsequent vaccination studies will test alternative delivery options and the long-term efficacy of the Δ*simA* mutant as a live attenuated vaccine in aquaculture.

## Materials and Methods

### Bacteria strains, culture, transformation, and DNA techniques

The WT virulent *S. iniae* strain K288, isolated from the brain of a diseased HSB at the Kent SeaTech (KST) aquaculture facility in Mecca, CA [9], served as a background for generation of the Δ*simA* and Δ*scpI* isogenic mutants. Additional *S. iniae* isolates used for comparative DNA sequence analysis are listed in Table 1. *S. iniae* was grown at 30°C (unless otherwise stated) in Todd-Hewitt broth (THB, Hardy Diagnostics) or on THB agar (THA). Enumeration of colony-forming units (CFU) was done through serial dilution of samples in PBS and plating on THA. β-hemolytic activity was assessed on sheep blood agar (SBA) plates (tryptic soy agar with 5% sheep red blood cells). For all assays, overnight cultures of *S. iniae* were diluted 1∶10 in fresh THB and grown to mid-log phase (OD_600_ = 0.40). *S. iniae* strains were rendered electrocompetent for transformation through growth in THB media containing 0.6% glycine following procedures described for GBS [82]; transformants were propagated at 30°C in THB with 0.25 M sucrose. Antibiotic selection was achieved with chloramphenicol (Cm) at 2 µg/ml or erythromycin (Erm) at 5 µg/ml. *Escherichia coli* used in cloning were grown at 37°C (unless otherwise stated), shaking, under aerobic conditions in Luria-Bertani broth (LB, Hardy Diagnostics) or statically on agar (LA). When necessary, *E. coli* were grown in antibiotics: ampicillin (Amp) at 100 µg/ml, spectinomycin (Spec) at 100 µg/ml, Erm at 500 µg/ml, or Cm at 20 µg/ml. Mach 1 chemically-competent *E. coli* (Invitrogen) and electrocompetent MC1061 *E. coli* used in transformations were recovered through growth at 30°C in S.O.C. media (Invitrogen). A PureLink™ Quick Plasmid Miniprep Kit (Invitrogen) was used to isolate plasmids propagated in *E. coli*. *S. iniae* genomic DNA was isolated using a Colony Fast-Screen™ Kit (EPICENTRE Biotechnologies) or an UltraClean DNA Isolation Kit (MoBio).

### Cell lines and culture conditions

The adherent CLC carp monocytic/macrophage cell line (European Collection of Cell Cultures no. 95070628) and the WBE27 white bass embryonic epithelial cell line (ATCC no. CRL-2773) [83] were grown at 28°C with 5% CO_2_. Cells were maintained in 125-ml tissue culture flasks in DMEM media (Gibco) containing 10% heat-inactivated fetal bovine serum (FBS, Gibco).

### Allelic exchange mutagenesis

Allelic exchange mutagenesis of *simA* (Fig. 1A) and *scpI* (Fig. 1B) with a chloramphenicol resistance gene, *cat*, was carried out as previously described for *S. iniae* [7]. A list of primers used to generate and confirm the allelic replacement mutants is provided (Table 3). PCR was used to amplify ∼1,000 bp of *S. iniae* chromosomal DNA fragments directly upstream and downstream of *simA* (primers 4+5, 6+7) and *scpI* (primers 10+11, 12+13), with primers adjacent to each gene constructed to possess 25 bp 5′-extensions corresponding to the 5′- and 3′- ends of the chloramphenicol acetyltransferase (*cat*) gene from pACYC [84], respectively. The upstream (Up) and downstream (Down) PCR products were then combined with a 660-bp amplicon of the complete *cat* gene (generated with primers 1+2) using fusion PCR (primers 4+7 for *simA*, 10+13 for *scpI*) [85]. The resultant PCR amplicon containing an in-frame substitution of *simA* and *scpI* with *cat* was subcloned into the Gateway entry vector pCR8/GW/TOPO (Invitrogen) and transformed into Mach 1 *E. coli* (Invitrogen). Plasmid DNA was extracted and a Gateway LR recombination reaction was performed to transfer the fusion PCR amplicon into the corresponding Gateway entry site of a temperature-sensitive knockout vector pKODestErm (a derivative of pHY304 [86] created for Gateway cloning), thereby generating the knockout plasmids pKOsimA and pKOscpI. The knockout constructs were introduced into WT K288 *S. iniae* by electroporation. Transformants were identified at 30°C by Erm selection then shifted to the nonpermissive temperature for plasmid replication (37°C). Differential antibiotic selection (Cm^R^ and Erm^S^) was used to identify candidate allelic exchange mutants. Targeted in-frame replacement of both genes was confirmed unambiguously by PCR reactions (primers 3+8 for *simA*, 9+14 for *scpI*) documenting the desired insertion of *cat* and absence of *simA* and *scpI* sequence in chromosomal DNA isolated from the final isogenic mutants, Δ*simA* and Δ*scpI*.

**Table 3:** Primers used to generate and confirm Δ*simA* and Δ*scpI* allelic mutants.

Number	Primer name	Sequence (5′–3′)
1	catF	atggagaaaaaaatcactggatataccacc
2	catR	ttacgccccgccctgccactcatcgcagta
3	simA−1063F	aggcagagaacatttcagacaag
4	simA−1015F	agtctgtttcaaacttgtcatg
5	simA−22cat27R	ggtatatccagtgatttttttctccatgtttagggttctccttattttc
6	cat635simA+25F	gcgatgagtggcagggcggggcgtaagctttcccttgcaaccttttcatag
7	simA+1016R	aagtacaaggatgctagccctg
8	simA+1152R	agatttcgggcaagctgccgttg
9	scpI−859F	agcagatcacattgttagtg
10	scpI−821F	tagcaccttcattagcagtc
11	scpI−18cat27R	ggtatatccagtgatttttttctccataatatattcctccaatag
12	cat635scpI+19F	gcgatgagtggcagggcggggcgtaaatcaaaaaaagaatgttcg
13	scpI+1044R	acaaaaattgctgagagttatg
14	scpI+1176R	tcttattggaacttatctgg

### Identification of M-like protein and C5a peptidase homologues

Short contigs generated from pyrosequencing (454 Life Sciences) of the *S. iniae* K288 genome were assembled using the Phred/Phrap/Consed suite (http://www.phrap.org/phredphrapconsed.html), resulting in 1865 contigs ranging in size from 51 bp to 22 kb. Without the need of further assembly, we used these contigs to build our *S. iniae* genome database that we used for BLAST searches. Using a local version of BLAST (version 2.2.14) [87], BLAST analysis of each contig against GAS M1 (GenBank Accession No. NC_002737) and M3 (GenBank Accession No. NC_004070) genomic sequences revealed the presence of putative M-like protein and C5a peptidase homologues. The contigs possessing hits for M-like protein and C5a peptidase genes were analyzed using Vector NTI software (Invitrogen) to assign open reading frames. Single-primer PCR [88] was used to sequence out from the contig ends in order to generate complete target gene sequences and provide at least 1,000 bp of flanking genomic sequence for use in allelic exchange mutagenesis. Finally, genomic regions containing *simA* and *scpI* were resequenced using standard BigDye sequencing techniques (Eton Bioscience Inc.) to confirm data generated in the initial sequencing efforts. Results from our K288 genomic analysis were compared with preliminary *S. iniae* (strain 9117) sequence data obtained from the BCM-HGSC website (http://www.hgsc.bcm.tmc.edu).

### Public reporting of sequence data

Sequences for *simA* and surrounding chromosomal genes for *S. iniae* strains K288 and 02161A were deposited in the GenBank database under accession numbers EU693238 and EU714186, respectively. Sequences for *scpI* and flanking genes in strain K288 were deposited under the accession number EU693239.

### Bioinformatic analyses

The amino acid sequences of all proteins were retrieved from the National Microbial Pathogen Data Resource (NMPDR) database (http://www.nmpdr.org/) by the use of the SEED similarity tool [89] and the NMPDR bidirectional best-hit engine [90]. For confirmation and completion of any missing sequences, the procedure was repeated by the use of BlastP and tBlastN algorithms [87] to search the non-redundant protein database (nr proteins) filtered to the genus *Streptococcus*. SignalP version 3.0 algorithm was used to screen the proteins sequences for Gram-positive leader peptides [91]. Several tools were used for motif finding, including InterPro [92], Pfam [93], in addition to FigFam [94] FASTA-formatted protein sequences were used as an input for the ClustalW software [95], [96] available as a part of the Biology Workbench Server, (http://workbench.sdsc.edu) [97]. Phylogenetic distances of the alignment results were calculated by Phylip analysis [98], and phylogenetic trees were drawn by DrawGram [97].

### Red blood cell hemolysis

Fresh, heparinized, whole HSB blood was diluted 1∶1 with HBSS (no Ca^2+^ or Mg^2+^) and 8 ml added to the top of a layered Percoll (Sigma) gradient containing 8 ml of 1.06, 1.07, and 1.08 g/ml solutions. The tube was centrifuged at RT for 30 min at 350×*g*. Red blood cells were taken from the bottom of the 1.08 g/ml density layer, washed three times in 20 volumes of PBS, and resuspended as a 2% solution (v/v). In a 96-well round bottom plate, mid-log cultures of bacteria were aliquoted in quadruplicate in volumes of 100 µl. Each well then received 100 µl of the 2% fish blood solution. Background lysis was measured in wells containing only blood cells and THB. Complete lysis was measured by wells containing blood cells, sterile THB, and 2 µl of Triton X-100. Plates were incubated at 30°C for 2 h then at 4°C for 2 h. Following centrifugation at 1,500×*g* for 5 min, 100 µl from each well was added to a new flat-bottom 96-well plate and the optical density was read at 405 nm in a microplate reader (Molecular Devices).

### Cell surface charge

In triplicate, overnight cultures of each *S. iniae* strain were diluted 1∶10 and grown to mid-log phase. Five ml of each culture was washed once in PBS prior to resuspension in 400 µl of MOPS buffer (pH 7.0). Next, 100 µl of a 5 mg/ml solution of cytochrome *c* (Sigma) was added. The solution was mixed thoroughly and incubated at room temp for 15 min. The bacterial suspension was pelleted (16,000×*g* for 5 min) and 200 µl of the supernatant was added to new flat-bottom 96-well plate. Controls included MOPS alone and MOPS with the same proportionate amount of cytochrome *c*. The amount of unbound cytochrome *c* was determined by absorbance of the supernatant at 530 nm.

### HSB virulence challenges

*In vivo* virulence attenuation of Δ*simA* and Δ*scpI* mutants was assessed in juvenile (∼15 g) HSB (*Morone chrysops*×*Morone saxatilis*) as previously described [7]. Groups of 10 fish per treatment group were injected intraperitoneally (IP) or intramuscularly (IM) in the dorsal muscle with 3×10^5^ CFU suspended in 50 µl of PBS. Fish were maintained at 24°C in aerated, 113-l flow-through tanks and monitored one week for survival. All Fish challenges were carried out in an AAALAC-certified facility following IACUC-approved protocols.

### Zebrafish virulence challenges

Adult zebrafish (*Danio rerio*, strain EKW) were challenged IM (n = 10 fish per treatment group) with 5×10^4^ CFU of Δ*simA*, Δ*scpI*, or WT K288 as previously described [40]. Mid-log phase bacteria were diluted in PBS and injected in 10-µl volumes using a 0.3 cc syringe with a 29-gauge needle. Fish were maintained at 28°C in recirculated, 10-l aquariums. Survival was monitored for one week post challenge.

### Antimicrobial peptide (AMP) killing assays

AMPs moronecidin [43], polymyxin B (Sigma), and CRAMP [99] were diluted in distilled H_2_O to 15, 600, or 160 µM, respectively. In a 96 well round bottom plate 10 µl of each AMP solution was added to 90 µl of THB containing ∼1×10^5^ CFU bacteria taken from a mid-log phase culture. The plate was incubated at 30°C. At each time point a 25-µl aliquot was removed, serially diluted in THB, and plated on THA. Survival was calculated by dividing surviving CFU from each time point by the starting CFU for each strain.

### Invasion and adherence assays

Invasion and adherence assays were performed in collagen-coated 96-well tissue culture plates (Nunc) using confluent monolayers of WBE27 white bass epithelial cells. Mid-log phase bacteria were centrifuged at 3,500×g for 5 min then washed once in PBS. Bacteria were then resuspended in DMEM containing 2% FBS and added to each well in 100 µl volumes to achieve a multiplicity of infection (MOI) of 5 (bacteria∶cells). Following centrifugation at 350×*g* for 10 min, the plate was incubated for 60 min at 28°C with 5% CO_2_. The cells were then washed twice with DMEM containing 2% FBS and incubated in fresh DMEM with 30 µg/ml penicillin (Invitrogen) and 300 µg/ml of gentamicin (Invitrogen) for 60 min to kill extracellular bacteria. Cells were then washed twice with DMEM containing 2% FBS and lysed by trituration in 100 µl of 0.025% Triton X-100 (Sigma). Surviving intracellular bacteria were quantified by plating serial dilutions of lysed cell supernatant on THA. The 30 min adherence assays were carried out in a similar manner except that no antibiotics were used and wells were washed five times with DMEM containing 2% FBS to remove non-adherent bacteria prior to trituration and enumeration of CFU.

### Macrophage survival assay

Monolayers of carp macrophages (CLC) were grown as described for the invasion and adherence assays. Bacteria were washed once in PBS then diluted in DMEM containing 2% FBS, added to the cells at an MOI of 0.05, and incubated at 28°C for 2, 4, or 18 h. Without washing, 25 µl of a 0.125% Triton X-100 solution was added to each well (0.025% final concentration). Cells were lysed and bacteria were plated as described above for invasion and adherence assays. Survival was calculated as a percentage of the input inoculum.

### HSB vaccine trials

Live attenuated vaccine challenges of Δ*simA* were carried out similar to the HSB virulence studies described above. Single groups of 25 HSB (∼21 g) were fin clipped to indicate treatment group and injected IP with a 100 µl volume containing 3×10^4^ or 3×10^6^ CFU of the Δ*simA* mutant or with PBS alone. Fish were held at 24°C for 2 weeks in 113-l aerated, flow-through tanks. Fish were cohabitated in a 1,071-l recirculating tank and held at 14-16°C for 1400 degree days (∼90 days total). Fish were then sorted by treatment group into 113-l challenge tanks and acclimated to 24°C over a period of 2 days. Each group was then challenged with a 100-µl IP injection of 5×10^5^ CFU of WT *S. iniae*. Survival was monitored for 2 weeks.

### Statistical analyses

Data analyses were performed using the statistical tools included with GraphPad Prism 5 (GraphPad Software, Inc.). *In vitro* assay data were analyzed using unpaired two-tailed t-tests. Fish infection survival data were analyzed using a Logrank Test. *P*<0.05 was considered statistically significant. *In vitro* assays were repeated three times (with equivalent results), in quadruplicate, and data presented (mean±standard error of the mean, SEM) are from a single representative assay. *In vivo* fish challenges were repeated twice with equivalent results and data from a single experiment are shown.

## Supporting Information

Figure S1 Full length amino acid sequence alignment among SiMA and M family proteins with highest similarity. Strain abbreviations: SIn-*S. iniae*, SPy-*S. pyogenes*, SUb-*S. uberis*, SEq-*S. equi*, and SDy-*S. dysgalactiae*
(24.65 MB TIF)

Figure S2 Amino acid alignment of ScpI with GAS and GBS C5a peptidases. ScpI shows high sequence similarity to the closest C5a peptidase homologues from GAS (ScpA, SPy Manfredo M5 strain) and GBS (ScpB, SAg A909 strain). ScpI possesses the conserved LPXTN Gram-positive surface anchor motif (dark line) as well as the Asp-His-Ser catalytic triad residues (asterisks), though proteolytic function of ScpI is unknown.
(13.22 MB TIF)
