# Supplementary figures and images for: Streptococcus iniae M-Like Protein Contributes to Virulence in Fish and Is a Target for Live Attenuated Vaccine Development
Source: PLoS One. 2008 Jul 30;3(7):e2824. doi: 10.1371/journal.pone.0002824 (PMC2483786; doi:10.1371/journal.pone.0002824)

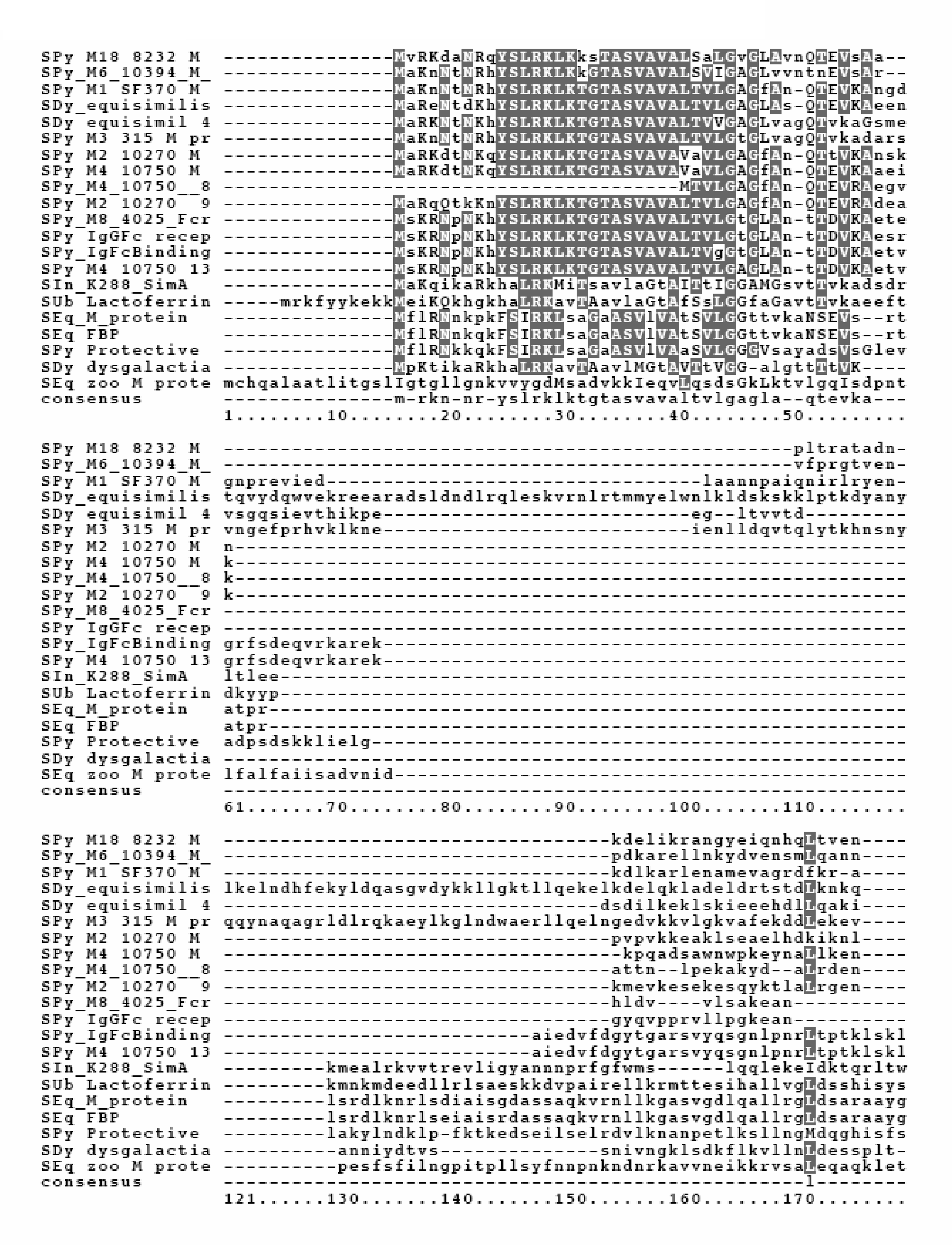

Supplement: Figure S1 — Full length amino acid sequence alignment among SiMA and M family proteins with highest similarity. Strain abbreviations: SIn-S. iniae, SPy-S. pyogenes, SUb-S. uberis, SEq-S. equi, and SDy-S. dysgalactiae (24.65 MB TIF) [file pone.0002824.s001.tif]

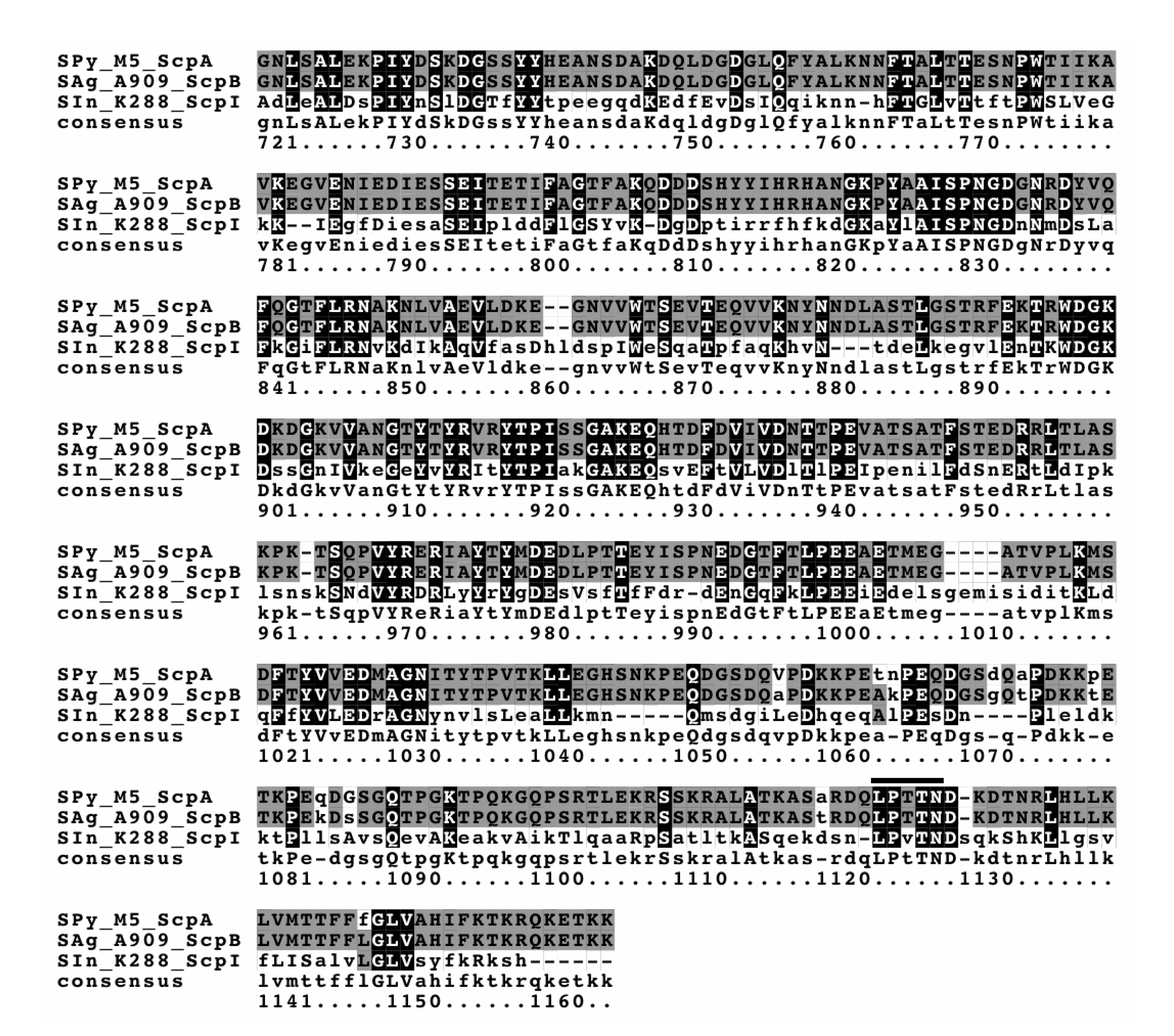

Supplement: Figure S2 — Amino acid alignment of ScpI with GAS and GBS C5a peptidases. ScpI shows high sequence similarity to the closest C5a peptidase homologues from GAS (ScpA, SPy Manfredo M5 strain) and GBS (ScpB, SAg A909 strain). ScpI possesses the conserved LPXTN Gram-positive surface anchor motif (dark line) as well as the Asp-His-Ser catalytic triad residues (asterisks), though proteolytic function of ScpI is unknown. (13.22 MB TIF) [file pone.0002824.s002.tif]
